# Feasting in fresh water: impacts of food concentration on freshwater tolerance and the evolution of food × salinity response during the expansion from saline into fresh water habitats

**DOI:** 10.1111/eva.12054

**Published:** 2013-03-04

**Authors:** Carol Eunmi Lee, Wynne E Moss, Nora Olson, Kevin Fongching Chau, Yu-Mei Chang, Kelsey E Johnson

**Affiliations:** 1 Center of Rapid Evolution (CORE), University of WisconsinMadison, WI, USA; 2 Research Office, The Royal Veterinary CollegeLondon, UK

**Keywords:** freshwater colonization, natural selection, osmoregulation, phenotypic plasticity, range limits, reaction norm, starvation resistance

## Abstract

Saline to freshwater invasions have become increasingly common in recent years. A key hypothesis is that rates of freshwater invasions have been amplified in recent years by increased food concentration, yet this hypothesis has remained unexplored. We examined whether elevated food concentration could enhance freshwater tolerance, and whether this effect evolves following saline to freshwater invasions. We examined physiological response to salinity and food concentration in a 2 × 2 factorial design, using ancestral brackish and freshwater invading populations of the copepod *Eurytemora affinis*. We found that high food concentration significantly increases low-salinity tolerance. This effect was reduced in the freshwater population, indicating evolution following the freshwater invasion. Thus, ample food could enable freshwater invasions, allowing subsequent evolution of low-salinity tolerance even under food-poor conditions. We also compared effects of food concentration on freshwater survival between two brackish populations from the native range. Impacts of food concentration on freshwater survival differed between the brackish populations, suggesting variation in functional properties affecting their propensity to invade freshwater habitats. The key implication is that high food concentration could profoundly extend range expansions of brackishwater species into freshwater habitats, potentially allowing for condition-specific competition between saline invaders and resident freshwater species.

## Introduction

The spread of invasive species continues unabated throughout the globe, with rates of invasions accelerating in many habitats throughout the world (Gaston et al. 2003; Solow and Costello 2004; Drake and Lodge 2007; Ding et al. 2008). However, of the large numbers of species that are introduced, comparatively few become successful as invaders (Williamson and Fitter 1996). While transport opportunity and ecological factors impact the frequency and success of introductions, ample evidence indicates that intrinsic properties of populations, both physiological and evolutionary, are also profoundly important in influencing invasive success (Lee 2002, 2010; Lee and Petersen 2003; Lee et al. 2003, 2007, 2011; Phillips et al. 2006; Colautti et al. 2010; Seebacher and Franklin 2011).

In aquatic habitats, salinity poses among the greatest challenges limiting invasions into novel habitats (Lee and Bell 1999), as a biogeographic boundary of ∼5 PSU (SI unit for salinity ≍ parts per thousand salinity) typically separates distributions of saline and freshwater invertebrate species (Khlebovich and Abramova 2000). Despite this formidable barrier, saline to freshwater invasions have been overrepresented among aquatic invaders, relative to their rate of transport (Jażdżewski 1980; Lee and Bell 1999; Ricciardi and MacIsaac 2000; Cristescu et al. 2003, 2004; May et al. 2006; Ricciardi 2006; Lee and Gelembiuk 2008; Keller et al. 2011). In fact, many species of brackishwater origin, such as the zebra mussel (*Dreissena polymorpha*), the quagga mussel (*Dreissena bugensis*), the waterhook flea (*Cercopagis pengoi*), as well as many amphipod species, now dominate many freshwater bodies throughout the world (Jarvis et al. 2000; Gelembiuk et al. 2006; May et al. 2006; Peyer et al. 2009; Strayer 2009; Zaiko et al. 2011). Why have brackishwater species become so successful as invaders into freshwater habitats?

Low salinity typically poses a serious barrier for species from saline habitats (Khlebovich and Abramova 2000), such that an evolutionary response has been found to accompany the transition from saline to freshwater habitats. For example, studies on the estuarine and salt marsh copepod *Eurytemora affinis* have revealed that freshwater invasions have been accompanied by evolutionary shifts in salinity tolerance, with an increase in freshwater tolerance and loss of high-salinity tolerance (Lee et al. 2003, 2007). In addition, this copepod has experienced evolutionary changes in ionic regulation following freshwater invasions (Lee et al. 2011, 2012). In the case of the zebra mussel *Dreissena polymorpha*, populations reside at ∼4–10 ppt salinities in their native Black and Caspian Sea ranges, whereas freshwater invading populations in Europe and North America have acquired low-salinity tolerance and tend to occur at salinities lower than 1 ppt (McMahon 1996; Karatayev et al. 1998). However, despite apparent adaptation to freshwater conditions, these invaders from brackish water, such as zebra mussels and the amphipod *Corophium curvispinum,* still tend to be inefficient ionoregulators in fresh water relative to long-term resident freshwater species (Taylor and Harris 1986; Dietz et al. 1996).

Eutrophic conditions have been proposed to promote invasions into freshwater habitats, due to the enormous productivity of invasive species and their competitive advantage under high resource conditions (Engelhardt 2011; Spaak et al. 2012). In particular, saline to freshwater invaders have the added burden of less efficient ionoregulatory capacities under freshwater conditions, and might be even more constrained by food concentration and high caloric intake to maintain ionic balance in freshwater environments (Taylor and Harris 1986; Dietz et al. 1996; Lee et al. 2011, 2012). In addition, brackishwater invaders have been found to possess strong preference for particular types of food following invasions into freshwater habitats. For example, suspension feeders of brackish origin, such as zebra mussels and the copepod *Eurytemora affinis*, selectively feed on algae high in particular long-chain polyunsaturated fatty acids, such as cryptophytes, and preferentially invade freshwater habitats harboring such a food source (Munawar and Munawar 1976; Makarewicz 1993; Vanderploeg et al. 1996; Lee 1999; Wacker et al. 2002; Wacker and von Elert 2004; Westerhoff et al. 2005; Naddafi et al. 2007; Basen et al. 2011).

Thus, this study tests the hypothesis that enhanced food concentration could increase freshwater tolerance, with the implication that such an effect would facilitate invasions by brackishwater invertebrates into freshwater environments. As freshwater habitats are often food-poor relative to estuarine environments (Lovejoy et al. 1993; Makarewicz 1993; Vincent et al. 1994; Martineau et al. 2004; Westerhoff et al. 2005), brackishwater invaders might often be constrained by food availability in freshwater habitats, as well as by low salinity. That is, high concentrations of appropriate food might enable saline populations to initially invade otherwise inhospitable freshwater environments, providing opportunities to subsequently evolve freshwater tolerance even when food concentrations are low.

Given the hypothesis of this study (stated above), the specific goals were to determine (i) whether food concentration influences low-salinity tolerance, (ii) whether the effect of food concentration varies at different salinities, and (iii) whether the effect of food concentration on freshwater tolerance has evolved following saline to freshwater invasions. In addition, we determined (iv) whether impacts of food concentration on freshwater survival differ between two distinct populations from the saline native range.

**Figure 1:**
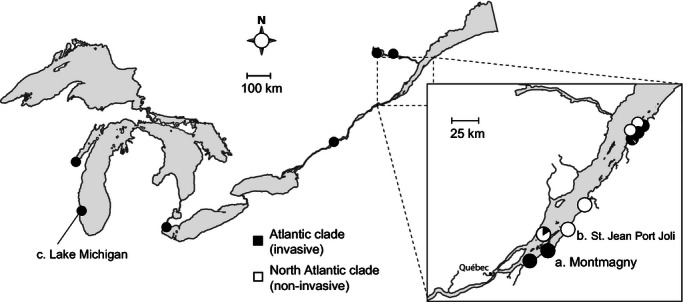
Map of the Great Lakes and St. Lawrence River system showing geographic distribution of *E. affinis* populations from the Atlantic and North Atlantic clades, based on sample collections from Winkler et al. (2008). Dots indicate locations of *E. affinis* collections, with the proportion of black (invasive Atlantic clade) and white (noninvasive N. Atlantic clade) colors in each dot indicating the proportion of animals from each clade sampled at each location. The inset provides a detailed map of the St. Lawrence estuarine transition zone and downstream regions of the lower estuary, showing two populations (a: Montmagny and b: St. Jean Port Joli) used in this study. Population genetic structure and haplotype frequencies within populations are presented in Winkler et al. (2008).

To address our goals, we used the copepod *Eurytemora affinis* (Poppe 1880), a brackishwater crustacean that has invaded freshwater habitats multiple times independently within the past few decades (Lee 1999; Winkler et al. 2008). We compared survival during development and development time between the ancestral saline (Fig. 1, site a) versus freshwater invading (site c) populations at (i) two salinities (fresh [75 μS/cm] and brackish [1 PSU]) and (ii) two food concentrations (∼700 and 14 000 cryptophyte algal cells/mL) in a 2 × 2 factorial design. In addition, the native range of the copepod *Eurytemora affinis* in the St. Lawrence estuarine zone is inhabited by two clades that overlap in distribution (Fig. 1), but only one clade has given rise to populations that could invade freshwater habitats (Fig. 1, Atlantic clade) (Lee 1999; Winkler et al. 2008). Thus, we compared freshwater survival across three food concentrations (∼700, 2800, 14 000 cryptophyte algal cells/mL) between saline populations from the invasive (site a) and noninvasive clades (site b) in the St. Lawrence estuary. For our experiments, we used the cryptophyte alga *Rhodomonas minuta* as the food source. We already know that this food type, rich in particular long-chain polyunsaturated fatty acids, is required for freshwater survival of such brackish invaders (Vanderploeg et al. 1996; Lee et al. 2003; Naddafi et al. 2007), such that in this study we tested the hypothesis that food quantity is important for freshwater survival by brackishwater invaders.

If we find that higher food concentration elevates freshwater survival, the implication would be that higher food concentration could potentially increase the extent to which brackish species could invade freshwater habitats. Significant food × salinity interaction effects on survival would indicate that food concentration has differential impacts at different salinities. In fact, if food concentration does indeed enhance low-salinity tolerance, we would expect that its ‘rescue’ effect would be greater at reduced salinities. Differences in the impact of food concentration on freshwater tolerance between the ancestral brackish and freshwater invading populations would indicate that this impact has evolved following the invasion into fresh water. Finally, differences in the effect of food concentration on freshwater survival between saline populations from the native range would suggest variation among native range populations in functional properties that might affect their propensity to invade freshwater habitats. Overall, significant effects of food concentration on freshwater survival would imply that anthropogenic nutrient inputs into freshwater habitats, and subsequent increases in primary productivity, could promote invasions by brackishwater species into freshwater environments.

## Materials and methods

### Population sampling

The copepod *E. affinis* first invaded the freshwater Great Lakes from the brackish St. Lawrence estuary in 1958 (Anderson and Clayton 1959), coincident with the opening of the St. Lawrence seaway. To analyze the physiological adaptations following this invasion, we compared the saline (ancestral) and freshwater (derived) populations of *Eurytemora affinis* within the St. Lawrence drainage system. Specifically, we examined an ancestral saline (brackish) population in the St. Lawrence estuary at Montmagny, PQ, Canada (Fig. 1a, 46° 58′ 45 N, 70° 33′ 21 W) and a freshwater invading population from Lake Michigan at Racine Harbor, WI, USA (Fig. 1c; 42°43′46″ N, 87°46′44″ W) from the invasive Atlantic clade. The brackish population at Montmagny in the St. Lawrence estuary resides in the upstream reaches of the estuarine transition zone, and has been identified as ancestral to populations in the Great Lakes based on genetic analyses (Winkler et al. 2008).

To compare responses between brackish populations within the native range in the St. Lawrence drainage, we included an estuarine population from the noninvasive North Atlantic clade from St. Jean Port Joli, PQ, Canada (Fig. 1b, 47° 12′ 35 N, 70° 15′ 36 W). Because the Atlantic and N. Atlantic clades are morphologically indistinguishable (Lee and Frost 2002), we confirmed clade (and population) identity by sequencing mitochondrial cytochrome oxidase I (COI) and 18S rRNA using PCR and DNA sequencing protocols outlined in previous publications (Lee 2000; Lee and Frost 2002).

While salinity of Lake Michigan is near 0 PSU (practical salinity units, SI unit for salinity ≍ parts per thousand salinity) conductivity, a more sensitive measure at lower salinities, is typically in the range of 300 μS/cm (St. Lawrence Centre 1996; Barbiero and Tuchman 2001). For the estuarine samples, surface salinity measurements taken at the time of collection were 0.1 PSU at Montmagny and 4 PSU at St. Jean Port Joli. While salinity differed between the populations at the time of collection, both populations occur in the estuarine transition zone, where salinity fluctuates spatially and temporally. Conductivities at these sites are appreciably higher than those of the Great Lakes. For example, at Montmagny conductivity ranged from 500 to ∼1600 μS/cm during a late summer month of 2000 (Roy 2002). In addition, the shallow bays around Montmagny and St. Jean Port Joli are within only a few kilometers of a deeper channel that has higher salinity at depth (d'Anglejan and Ingram 1976).

Fresh water is often defined as salinities below 0.5 ppt (parts per thousand ≍ PSU), whereas brackish water is typically defined as salinities within the range of 0.5–30 ppt (Dahl 1956). The term ‘saline’ includes both brackish and marine water. The convention of ‘fresh water’ as a noun and ‘freshwater’ as an adjective is used throughout this article.

### Testing effects of food concentration on freshwater survival in saline versus freshwater populations

We performed a common-garden reaction norm experiment to examine effects of food concentration and salinity on survival and development time of saline (Fig. 1a, St. Lawrence at Montmagny) and freshwater (Fig. 1c, Lake Michigan) populations of *E. affinis*. In a common-garden experiment, different populations are reared at a common environment to remove effects of environmental acclimation and reveal genetically based differences between populations. In a reaction norm experiment, populations are reared across environments to reveal the plastic phenotypic responses of genotypes to the different environmental conditions (or the ‘reaction norms’) (Schlichting and Pigliucci 1998). A common-garden reaction norm experiment, where different populations are reared at a common set of distinct environments, uncovers the genetically-based differences between populations in response to a set of environments. We determined response to food and salinity in a 2 × 2 factorial design, using two salinities (freshwater 75 μS/cm and brackishwater 1 PSU) and two food concentrations (700 and 14 000 cells/mL). Freshwater bodies typically register around 0 PSU on the salinity scale, such that conductivity is typically used to measure ionic concentrations at the lower salinities.

The ‘high’ salinity (1 PSU) treatment was chosen to resemble conditions where the population at Montmagny is found, in the relatively upstream regions of the St. Lawrence estuary. Brackish 1 PSU water was prepared by mixing Lake Michigan water (∼300 μS/cm conductivity, 0 PSU salinity) with Instant Ocean®. The ‘low’ salinity treatment was a mixture comprising of 25% Lake Michigan water and 75% deionized water. This mixture was chosen to achieve a conductivity of ∼75 μS/cm, at the lower end of the natural freshwater range. All water was filtered through 0.22 μm mesh to remove bacteria. To deter bacterial growth, the antibiotic Primaxin was administered to all treatments at a concentration of 20 mg/L.

For the food treatments, we used the freshwater cryptophyte alga *Rhodomonas minuta*, one of the more abundant phytoplankton species found in the Great Lakes (Reuter 1979; Makarewicz 1993), and one that tends to occur in eutrophic lakes (Moustaka-Gouni 1996). *Rhodomonas minuta* occurs in the habitats of the both the brackish and freshwater populations used in this study (Moustaka-Gouni 1996; Hudon 2000; Barbiero and Tuchman 2001). Other species of *Rhodomonas* that are consumed by populations of *E. affinis* also occur in the St. Lawrence drainage (Moustaka-Gouni 1996; Roy et al. 1996; Barbiero and Tuchman 2001). Food concentrations were selected to resemble those of the natural habitats of *E. affinis*. The concentration of the low food treatment (700 cells/mL) was within the range found for *R. minuta* in the Great Lakes, of 250–1500 cells/mL (Makarewicz 1993; Barbiero and Tuchman 2001). Likewise, the concentration of the high food treatment used in this study (14 000 cells/mL) was on par with phytoplankton abundance in the St. Lawrence estuarine zone (∼10 000 to >200 000 cells/mL, estimated from chlorophyll *a* measurements) (Lovejoy et al. 1993; Vincent et al. 1994; Martineau et al. 2004).

Prior to placing animals in the food and salinity treatments, juveniles were taken from laboratory cultures, reared to adulthood, mated, and allowed to produce eggs at a common salinity of 0.5 PSU. A common salinity of 0.5 PSU was used for both populations to minimize effects of maternal environment and acclimation to different native salinities, prior to placing the eggs into the four experimental treatments (next paragraph). We transferred juveniles rather than larvae (nauplii) to 0.5 PSU, because transfer of larvae would induce high mortalities and impose selection (Lee et al. 2003, 2007).

Once egg clutches were produced, we divided each clutch across four treatments (low food & low salinity, low food & high salinity, high food & low salinity, and high food & high salinity), with 2–5 eggs per treatment vial. Large clutches were divided 8 ways, with 2 replicates per clutch at each treatment. We used clutch means for all statistical analyses (see below). We regarded each full-sib clutch as a distinct genotype, as the clutches arose from distinct male × female crosses. The common-garden experiment was performed in two blocks, in August 2010 (with 8 St. Lawrence-Montmagny clutches, 6 Lake Michigan clutches) and December of 2010 (with 8 St. Lawrence-Montmagny clutches, 9 Lake Michigan clutches). The experiment was performed at 12°C, on a 15L:9D photoperiod. Visual inspections of the vials were performed daily or every other day to assess the number and developmental stage of individuals. We measured percentage hatching, survival from hatching to metamorphosis, and development time from hatching to metamorphosis as measures of fitness. Metamorphosis was defined as the transition between the nauplius VI and copepodid I stages. Hatching to metamorphosis tends to be the life-history stage most subjected to high mortality due to high- or low-salinity stress (Lee and Petersen 2003; Lee et al. 2007).

### Statistical comparison of saline versus freshwater populations

We examined effects of *Food Concentration* (700 vs 14 000 cells/mL)*, Salinity* (fresh [75 μS/cm] vs brackish [1 PSU])*, Population* (St. Lawrence at Montmagny versus Lake Michigan), *Clutch* and interactions between these factors on survival and development time from hatching to metamorphosis. We analyzed survival data in a generalized linear mixed-model framework using the glmer procedure (and glm when we included only fixed effects) in the lme4a package of R, and development time data in a linear mixed-model framework using the lmer procedure in the lme4 package of R (R Development Core Team 2008). We treated survival data as binary and the models used a logit link function. Fixed effects included *Food Concentration, Salinity, Population, Block* (date of experiment), *Food* × *Salinity, Population* × *Food Concentration, Population* × *Salinity,* and *Population* × *Food Concentration* × *Salinity*. Random effects included *Clutch* (proxy for genotype), random slope for *Food Concentration* (with respect to *Clutch*), and random slope for *Salinity* (with respect to *Clutch*). To determine significance of effects of each factor on survival or development time, we performed likelihood-ratio tests to compare the fit of full models relative to those with each factor excluded.

After examining the effects of each factor on survival or development time, we determined pair-wise differences between saline versus freshwater populations at each food and salinity treatment. We determined statistical significance between the populations at each treatment using the Wilcoxon rank sum test for percentage survival for clutches and Welch's two-sample *t*-test for average development time of clutches (using R). In addition, to further examine effects of food concentration on freshwater survival, we determined whether differences between survival at high- and low food concentrations differed significantly between salinity treatments and between populations using the Wilcoxon rank sum test in R. These comparisons were performed as *a priori* planned comparisons, rather than comparisons of all pairwise combinations, such that multiple testing correction was not required (Sokal and Rohlf 1995).

### Testing effects of food concentration on freshwater survival in saline populations from the native range

Our goal was to determine whether food concentration had differential effects on freshwater tolerance in two saline (brackish) populations from the native range in the St. Lawrence estuary. We reared one population each from invasive (Fig. 1a, St. Lawrence at Montmagny) and noninvasive clades (Fig. 1b, St. Lawrence at St. Jean Port Joli) across three food concentrations (700, 2800, 14 000 algal cells/mL, using *Rhodomonas minuta*) in fresh water (0 PSU, Lake Michigan water) and measured survival and development time.

Prior to the common-garden experiment, the populations were cultured at a common salinity of 5 PSU in the laboratory for approximately 28 months. During this period, we fed laboratory cultures a mixture of the cryptophytes freshwater *Rhodomonas minuta* and saline *R. salina* three times per week. For laboratory cultures and experiments, we maintained populations at 12°C, on a 15L:9D photoperiod, with 20 mg/L Primaxin to prevent bacterial infections.

We performed reaction norm experiments by dividing eight full-sibling egg sacs across the three food concentration treatments. Each full-sib clutch was regarded as a distinct genotype, as they arose from independent female × male matings. For each food level, we placed five eggs per clutch in vials with ∼0 PSU Lake Michigan water, fed the animals daily, and observed the vials every other day. The experiment was performed in two blocks, where the second half of the experiment was started 2 weeks after the first half. We recorded survival in terms of percentage hatching from egg, percentage survival from hatching to metamorphosis, and percentage survival from metamorphosis to adult. We defined the developmental stages as follows: (i) metamorphosis, as the transition between the nauplius VI and copepodid I stages, and (ii) adult (copepodid VI stage), when males developed geniculate right antennules, and when females developed large wing-like processes on the posterior end of their prosomes (body).

### Statistical comparison of saline populations from the native range

We used a tri-variate sequential ordinal probit model (Albert and Chib 2001) to analyze effects of food concentration on freshwater survival for the two saline populations (see previous section). This ordinal probit model is a liability threshold model where the liability is the predisposition for survival. We used the model to (i) assess the effects of fixed factors (i.e., *Population, Food Concentration, Population* × *Food* interaction) and random factors (i.e., *Clutch, Clutch* × *Food*) on survival, (ii) estimate genetic correlations between survival at the three food concentrations, and (iii) estimate differences in survival between the two populations at each food concentration. We did not analyze effects of the factors on development time, as there was no survival to adulthood for the noninvasive population at low food concentration (Fig. 5C). This ordinal probit model is a superior method for analyzing survival data with sequential life-history stages because it accounts for the discrete nature of survival and for the cumulative survival at each life-history stage. Survival data consisted of three life-history stages. We coded observations as 0 (if egg did not hatch), 1 (if hatched), 2 (if survived to metamorphosis), and 3 (if survived to adult). The probability model can be written as:


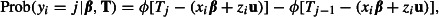


where *j* = 0, 1, 2, 3 indexes the category to which the observation belongs, Φ(·) is the standard cumulative normal density function, and **T** = [T_0_, T_1_, T_2_, T_3_]’ is the vector of unknown thresholds. The thresholds must satisfy -∞ < T_0_ ≤ T_1_ ≤ T_2_ < T_3_ = ∞. The first threshold T_0_ is set to zero, because the parameter cannot be identified in a probit analysis, leaving T_1_ and T_2_ as the only unknown thresholds. In the statistical model, **β** includes effects of *Population, Food Concentration,* and their interaction. **u** is a vector of random effects of *Clutch,* within *Population* and *Food Concentration,* and also *Clutch* × *Food*. We used likelihood-ratio tests to assess whether each of the fixed and random factors had significant effects on survival using the procedure NLMIXED in SAS (version 9.0) (SAS Institute Inc. 2003). We compared survival probabilities between the two populations using the ‘ESTIMATE’ statement in NLMIXED. This function computes t statistics, *P*-values, and confidence limits using approximate standard errors calculated by the delta method (Billingsley 1986; SAS Institute Inc. 2003). In addition, we used the Wilcoxon rank sum test (reported exact test *P*-value in SAS) to test for differences in mean survival of clutch between the two populations at each food concentration for each life-history stage.

## Results

### Response to food concentration and salinity in saline versus freshwater populations

**Figure 2:**
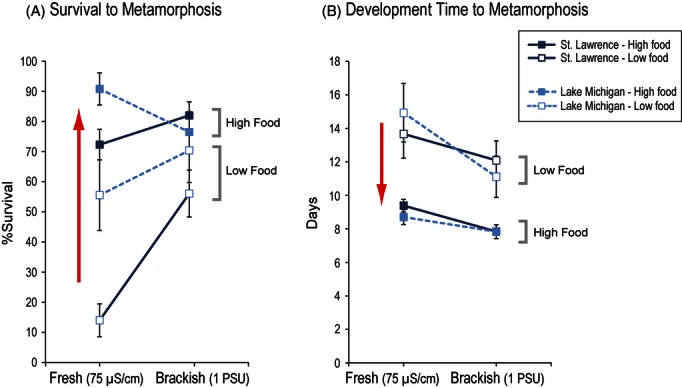
Beneficial impacts of higher food concentration on survival and development time for saline (Fig. 1a, St. Lawrence at Montmagny) and freshwater (Fig. 1c, Lake Michigan) populations. Survival and development time are shown for saline and freshwater populations reared at two food concentrations (700 and 14 000 cells/ml) and two salinities (fresh [75 μS/cm] and brackish [1 PSU]). Graphs show mean (A) survival from hatching to metamorphosis for 8-14 clutches per treatment and (B) development time from hatching to metamorphosis for 5-14 clutches per treatment. Error bars represent standard errors. Red arrows indicate the direction of impact of higher food concentration. Reaction norms for survival of individual clutches are shown in Fig. 3.

**Figure 3:**
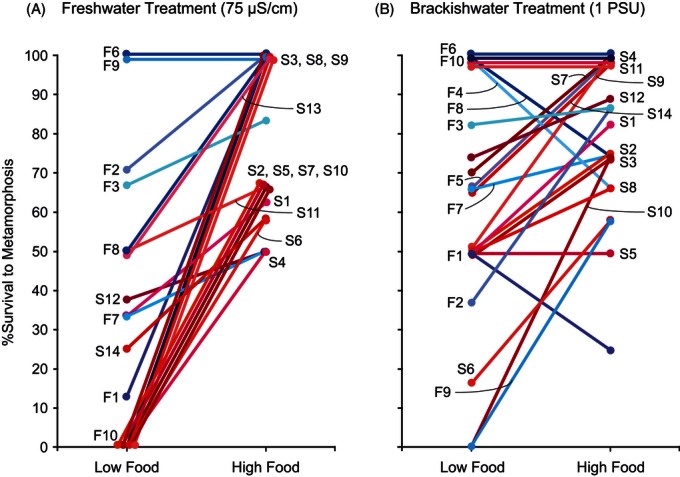
Impact of food concentration on survival, showing reaction norms of individual clutches reared at (A) low salinity (fresh water, 75 μS/cm) and (B) higher salinity (brackish water, 1 PSU). Colored lines in the graphs represent survival from hatching to metamorphosis of individual clutches from saline (red lines, St. Lawrence at Montmagny) and freshwater populations (blue lines, Lake Michigan) in response to low (700 cells/mL) and high food concentrations (14 000 cells/mL). Individual reaction norms are shown for 8-10 clutches from the freshwater population (blue, F1-F10) and 13-14 clutches from the saline population (red, S1-S14).

**Figure 4:**
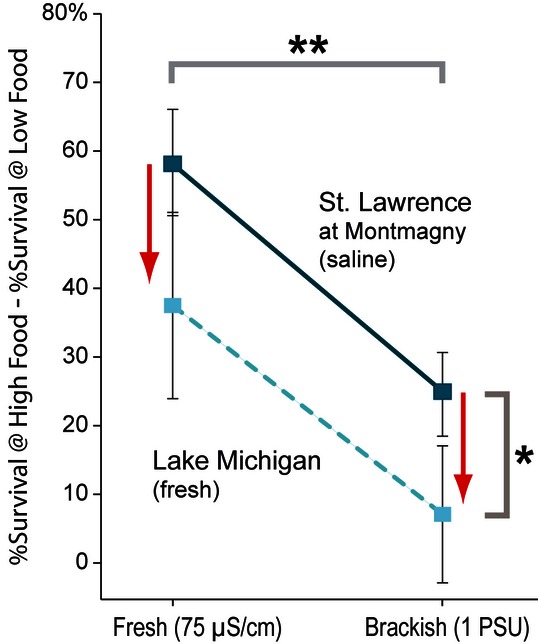
Impact of food concentration on survival to metamorphosis, showing differences in survival between high- and low food concentrations for saline (St. Lawrence at Montmagny) and freshwater populations (Lake Michigan) at two salinities (fresh [75 μS/cm], brackish [1 PSU]). Values are mean differences in survival between high- and low food concentration for each clutch, for 8-14 clutches ± SE. Graph shows that higher food concentration had a greater impact on survival at low salinity (75 μS/cm) than at high salinity (1 PSU) (Wilcoxon's *W* = 392, *P* = 0.0016). In addition, higher food concentration enhanced survival to a greater extent for the saline population (dark line) than for the freshwater population (dashed light line) (Wilcoxon's *W* = 334.5, *P* = 0.034). Red arrows indicate direction of evolution from saline to freshwater populations.

Our goals were to determine whether high food concentration could enhance freshwater survival and whether this effect could evolve following saline to freshwater invasions. We measured survival and development time of ancestral saline and derived freshwater populations in response to food concentration (700 and 14 000 cells/mL) and salinity (fresh water 75 μS/cm, brackish 1 PSU) in a 2 × 2 factorial design. We applied a mixed-model approach to determine the effects of the factors *Food Concentration, Salinity, Population, Clutch,* and interactions among these factors on survival and development time (see Materials and methods; Table 1). *Food Concentration* had highly significant effects on both survival and development time for both saline and freshwater populations (Figs 4; Table 1, first factor). Higher food concentration did show significant beneficial effects, in increasing survival (Fig. 2A, Fig. 3; St. Lawrence at Montmagny: Wilcoxon's *W* = 659, *P* < 0.0001; Lake Michigan: Wilcoxon's *W* = 268.5, *P* = 0.05) and reducing development time (Fig. 2B; St. Lawrence at Montmagny: Welch's *t* = −4.15, df = 18.51, *P* = 0.00057; Lake Michigan: Welch's *t* = −4.00, df = 18.44, *P* = 0.00081). The factor *Salinity* alone did not show overall significant effects on survival (Table 1A, second line; Fig. 2A), but did show significant effects on development time, which was reduced at the higher salinity (Table 1B; Fig. 2B).

**Table 1:** Effects of multiple factors on survival (using glmer or glm in R) and development time (using lmer in R) of saline and freshwater populations (Fig. 1a and 1c). Results show effects of fixed factors of *Population* (St. Lawrence at Montmagny versus Lake Michigan), *Food Concentration* (700 vs 14 000 cells/mL), *Salinity* (fresh [75 μS/cm] vs brackish [1 PSU]), *Block* (date of experiment), *Population* × *Food, Population* × *Salinity,* and *Food* × *Salinity,* as well as random effects of *Clutch*, random slope of *Food Concentration* and random slope of *Salinity*

	A. Survival		B. Development Time	
		
	Chi-square (DF)	*P*-value	Chi-square (DF)	*P*-value
Fixed Factors				
*Food Concentration*	15.72 (1)	**7.35** × **10**^**−5**^	73.82 (1)	**8.57** × **10**^**−18**^
*Salinity*	0.161 (1)	0.688	21.11 (1)	**4.32** × **10**^**−6**^
*Population*	25.21 (1)	**5.14** × **10**^**−7**^	0.289 (1)	0.591
*Block*	3.19 (1)	0.0743	0.099 (1)	0.753
*Food Concentration × Salinity*	5.54 (1)	**0.0186**	8.31 (1)	**0.00393**
*Population × Food Concentration*	1.76 (1)	0.184	0.016 (1)	0.900
*Population × Salinity*	11.84 (1)	**0.000579**	0.329 (1)	0.566
Random Factors				
*Clutch* (relative to model with fixed effects only)	0.177 (1)	0.674	33.51 (1)	**7.08** × **10**^**−9**^
Random Slope for *Food Concentration* (with respect to *Clutch*; *Clutch* × *Food*)	0.168 (3)	0.983	46.52 (3)	**4.40** × **10**^**−10**^
Random Slope for *Salinity* (with respect to *Clutch; Clutch × Salinity*)	0.758 (3)	0.860	0.722 (3)	0.868

Chi-square values, along with degrees of freedom (DF), and *P*-values are shown for likelihood-ratio tests between full models and those with each factor removed. Significant effects (< 0.05) that improve the model are shown in bold.

More importantly, we were more interested in the effects of food concentration across the different salinities (i.e., *Food* × *Salinity*) rather than the effects of *Food Concentration* or *Salinity* alone. If food concentration were important for energetic or ion-regulatory functions, we would expect this factor to have greater impacts at the lower salinity treatment (fresh water, 75 μS/cm). Indeed, we did find significant effects of a *Food* × *Salinity* interaction on survival and development time (Table 1, 5th factor), indicating that food had differing effects at the different salinities. In fact, higher food concentration did prove to be more critical for enhancing survival at the lower salinity (fresh water, 75 μS/cm) than at the higher salinity treatment (brackish, 1 PSU) (Figs 2A and 3; Wilcoxon's *W* = 392, *P* = 0.0016). This effect was apparent when examining the mean reaction norm slopes across salinities (Fig. 2A,B), as well as the reaction norms for individual clutches (genotypes) (Fig. 3). The beneficial impact of high food concentration on survival appeared more consistent and marked at the freshwater treatment (Fig. 3A) than at the higher salinity treatment (Fig. 3B). The greater impact of food concentration at lower salinity could be viewed more explicitly in Fig. 4, which plots differences in survival between high- and low food concentrations for the saline (St. Lawrence at Montmagny) and freshwater (Lake Michigan) populations. Higher values on the vertical axis indicate greater beneficial impacts of food concentration on survival. Significantly higher values at the lower salinity (fresh water, 75 μS/cm; Fig. 4, left values) than at higher salinity (1 PSU; Fig. 4, right values) indicate that higher food concentration increased survival to a greater extent at the lower salinity (75 μS/cm).

The significant effect of *Population* (Table 1, 3rd factor) indicates evolutionary shifts in response from the ancestral saline (Fig. 1a, St. Lawrence at Montmagny) to the freshwater invading populations (Fig. 1c, Lake Michigan). *Population* had significant effects on survival (Table 1A, 3rd factor; Fig. 2A), but not on development time (Table 1B, 3rd factor; Fig. 2B). Pairwise population comparisons revealed significant differences in survival between the saline and freshwater populations in response to both food concentration (Fig. 4) and salinity (Table 2A). The freshwater population showed significantly higher survival at the low food concentration (Wilcoxon's *W* = 136.5, *P* = 0.0067) and at low salinity (Table 2A, Fig. 2A), relative to the saline population (see next paragraphs). Pairwise population comparisons showed no significant differences in development time (Table 2B).

**Table 2:** Pairwise population comparisons of survival and development time, between saline (St. Lawrence at Montmagny) and freshwater (Lake Michigan) populations of *E. affinis,* in response to food concentration and salinity treatments. Survival was measured as percentage survival from hatching to metamorphosis, whereas development time was number of days from hatching to metamorphosis. Treatments consisted of combinations of two food concentrations (700 and 14 000 cells/mL) and two salinities (fresh [75 μS/cm] vs brackish [1 PSU])

Food concentration (cells/mL) Salinity	(a) 700 Fresh	(b) 700 Brackish	(c) 14 000 Fresh	(d) 14 000 Brackish
**A. % Survival**				
St. Lawrence (saline)	13.99 ± 5.45 (14)	56.09 ± 7.79 (13)	72.32 ± 5.09 (14)	82.02 ± 4.51 (14)
Lake Michigan (fresh)	55.56 ± 11.72 (9)	70.42 ± 10.62 (10)	90.83 ± 5.34 (10)	76.52 ± 6.79 (11)
Wilcoxon's *W*	19	46.5	35	87
*P*-value	**0.0042**	0.253	**0.033**	0.594
**B. Development time (days)**				
St. Lawrence (saline)	13.67 ± 1.44 (5)	12.09 ± 1.16 (12)	9.39 ± 0.37 (14)	7.84 ± 0.20 (14)
Lake Michigan (fresh)	14.93 ± 1.74 (8)	11.11 ± 1.24 (9)	8.71 ± 0.44 (10)	7.83 ± 0.41 (11)
Welch's *t* (DF)	−0.0557 (10.92)	0.576 (18.05)	1.176 (19.47)	0.022 (14.56)
*P*-values	0.589	0.572	0.254	0.983

Values for survival and development time are mean ± SE, with sample size in parentheses. Statistical significance between the populations at each treatment was determined using the Wilcoxon rank sum test for survival and a Welch two-sample *t*-test for development time data. Significant values (*P* < 0.05) are shown in bold. As these comparisons were performed as *a priori* planned comparisons, and not comparisons of all pairwise combinations, they did not require multiple testing correction (Sokal and Rohlf 1995).

Most notably, there were evolutionary shifts in the impact of food concentration from the saline to freshwater populations, where the beneficial impact of high food concentration on survival declined in the freshwater derived population relative to its saline ancestor (Fig. 4). In an *a priori* comparison between the saline and freshwater populations, the beneficial effect of higher food concentration on survival (high food survival – low food survival; Fig. 4) was significantly greater for the saline population (Fig. 4, dark line; Mean difference = 42.1% ± 0.059 SE) than for the freshwater population (Fig. 4, light dashed line; mean difference = 20.6% ± 0.087 SE) (Wilcoxon's *W* = 334.5, *P* = 0.034). That is, higher food concentration had a significantly greater positive impact on survival for the saline population than for the freshwater population, indicating that the saline ancestral population was more dependent on higher food concentration for survival. This evolutionary shift was evident from the downward shift in the curves in Fig. 4, from the saline population (dark line) to the freshwater population (light dashed line). The lack of difference in slope between the saline and freshwater populations in Fig. 4 indicated that the relative impact of food on survival at different salinities did not shift between populations, and was consistent with a lack of a significant *Population* × *Food Concentration* × *Salinity* effect on survival (using glm in R; *P* = 0.65).

The *Population* × *Food* interaction had no significant effect on survival or development time (Table 1, 6th factor). This result might appear to contradict the significantly greater impact of high food concentration on the saline population than on the freshwater population (previous paragraph). However, statistical tests that compare the differences in survival probabilities directly can be statistically significant when tests that compare differences in log survival odds are not, due to nonlinearity in the logistic transformation between probabilities and natural log of survival odds (Mendenhall and Sincich 2003). In this particular case, relatively large changes in survival probabilities (42% to 21%) corresponded to small changes when transformed to log odds (i.e., change between food levels of about 1.6 for both saline and freshwater populations).

The saline and freshwater populations showed significant differences in survival in response to salinity, evident in the differences in reaction norm slopes between the populations (Figs 2A and 3) and the significant *Population* × *Salinity* interaction for survival (Table 1A, 7th factor). The populations showed no significant difference in development time in response to salinity (Fig. 2B; Table 1B). Pairwise population comparisons showed that the freshwater Lake Michigan population had significantly higher survival than the saline St. Lawrence population at the lower salinity (75 μS/cm) (Table 2A, Columns a and c; Fig. 2A), but not at the higher salinity (1 PSU) (Table 2A, Columns b and d). This result indicates that the freshwater population evolved higher tolerance of low-salinity conditions, which would be consistent with adaptation to freshwater conditions following freshwater invasions.

The random factor of *Clutch*, the random slope for *Food* (i.e., *Clutch* × *Food*), and the random slope for *Salinity* (i.e., *Clutch* × *Salinity*) had no significant effects on survival (Table 1A). In contrast, the random factor of *Clutch* and the random slope for *Food* (i.e., *Clutch* × *Food*) did show significant effects on development time (Table 1B). As the full-sib clutches represent distinct genotypes, the significant effect of *Clutch* reveals the presence of genetic variation in development time. The significant *Clutch* × *Food* interaction indicates that clutches differed significantly in development time across different food concentrations (i.e., significant phenotypic plasticity).

*Block* effects (Table 1, 4th factor) were not significant for either survival or development time. This result indicated that different blocks (different experimental dates) did not yield significantly differing results and could be pooled for analyses.

### Effects of food concentration on freshwater survival in saline populations from the native range

Our goal was to determine whether two brackish populations from the native range in the St. Lawrence drainage (one each from invasive and noninvasive clades, Fig. 1a,b) differed in the impact of food concentration on freshwater survival. We used an ordinal probit model, which accounts for cumulative survival across life-history stages, to examine the effects of *Food Concentration, Population, Clutch*, and interactions among these factors on freshwater survival for the two brackish populations (see Materials and methods). Cumulative survival is a more relevant measure of survival, as survival across all life-history stages would affect population persistence in the wild.

**Figure 5:**
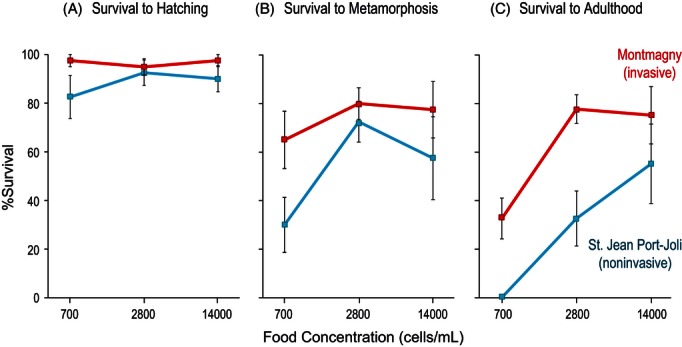
Survival in response to food concentration at 0 PSU (lake water) for brackish populations from the native range, from the invasive (Fig. 1a, Montmagny, red) and noninvasive (Fig. 1b, St. Jean Port Joli, blue) clades. Survival is shown in terms of (A) percentage hatching (B) percentage metamorphosis from those that hatched, and (C) percentage that developed to adulthood from those that metamorphosed. Graphs show mean clutch survival to adulthood ± SE across three food concentrations for 8 clutches per population. Statistical significance for the comparisons is shown in Table 4.

We used likelihood-ratio tests to determine significance of fixed and random factors in the ordinal probit model on freshwater survival (Table 3). Because the effect of *Population* × *Food* was not significant (Table 3, third factor, *P* = 0.95), we removed it from the full model when testing for effects of all other factors, except when testing for the effect of the random factor of *Clutch*. We found significant effects of *Food Concentration* on freshwater survival (Table 3, first factor, *P* = 0.0026), as evident in Fig. 5. *Population* also had significant effects on freshwater survival (Table 3, second factor, *P* = 0.012), revealing differences in response between the two brackish populations (Fig. 5). The significant effect of *Clutch* (Table 3, fourth factor, *P* = 0.005) indicated significant genetic effects on freshwater survival, whereas the significant effect of *Clutch* × *Food* (Table 3, fifth factor, *P* = 0.0003) revealed genetic variation in response to *Food Concentration* (i.e., genetic variation in plasticity, or reaction norms, in response to food concentration). The estimated genetic variance and broad sense heritability for liability to survival were 0.61 and 0.38, respectively.

**Table 3:** Effects of multiple factors, in an ordinal probit model (see Materials and methods), on survival of two brackish populations from the St. Lawrence estuary from invasive and noninvasive clades (Fig. 1a,b) reared at three food concentrations in fresh water (0 PSU). We used likelihood-ratio tests to determine significance of fixed factors of *Population* (Montmagny versus St. Jean Port Joli), *Food Concentration* (700, 2800, 14 000 cells/mL), *Population* × *Food*, and random factors of *Clutch*, random slope of *Food Concentration* (with respect to *Clutch*, or *Clutch* × Food). Because effects of *Population* × *Food* were not significant, we removed it from the full model when testing for effects of all other factors, except for *Clutch* (which was tested against a fixed effects only model)

	Chi-square (DF)	*P*-value
Fixed Factors		
*Food Concentration*	11.9 (2)	**0.0026**
*Population*	6.3 (1)	**0.0121**
*Population × Food*	0.1 (2)	0.951
Random Factors		
*Clutch*	8.0 (1)	**0.0047**
*Clutch × Food*	18.9 (3)	**0.0003**

Chi-square values, along with degrees of freedom (DF), and *P*-values are shown for likelihood-ratio tests between full models and those without a given factor. Significant effects (< 0.05) that improve the model are shown in bold.

For the full model, which included all factors in Table 3 and allowed for different genetic variances for each food concentration and different genetic correlations between survival at different food concentrations, the iterative fitting algorithm did not converge. This lack of convergence possibly arose from the small number of observations and insufficient information to estimate all 6 parameters in the (co)variance matrix. Thus, we instead used a reduced model that assumed the same genetic variance across food concentrations, while allowing for different genetic correlations between survival at the three food concentrations.

When we estimated genetic correlations between survival at the three food concentrations, we found a strong positive genetic correlation of 0.85 between survival at low and medium food concentrations (*P* = 0.014). This result indicates that selection for survival at the low food concentration (700 algal cells/mL) would favor survival at the medium food concentration (2800 algal cells/mL), and *vice versa*. On the other hand, we found negligible genetic correlations between low and high (−0.12, *P* = 0.71), as well as medium and high food concentrations (0.07, *P* = 0.82), indicating that selection favoring survival at the low to medium food concentrations (700–2800 algal cells/mL) would not select for survival at the high food concentration (14 000 algal cells/mL), and *vice versa*.

When using the ordinal probit model to compare pairwise differences in freshwater survival between the brackishwater populations, the invasive clade population (Fig. 1a, Montmagny) showed significantly higher survival to juvenile and adult stages across all food concentrations, relative to the noninvasive clade population (Fig. 1b, St. Jean Port Joli) (Table 4). However, when we examined survival at each separate life-history stage (i.e., to hatching, metamorphosis, and adult), the invasive clade population showed significantly higher survival to the juvenile stage at the lowest food concentration (700 cells/mL), relative to the noninvasive clade population (Table 4B, Wilcoxon rank sum test). The invasive clade population also showed higher survival to the adult stage at the two lower food concentrations (700 and 2800 cells/mL) than the noninvasive clade population (Table 4C, Wilcoxon rank sum test). Thus, these results revealed significant differences in freshwater survival between the brackish populations at the lower food range. Most notably, the noninvasive clade population showed a complete lack of survival to adulthood at the lowest food concentration (Fig. 5C).

**Table 4:** Pairwise population comparisons of freshwater survival across three food concentrations between two populations of *E. affinis* from the saline native range in the St. Lawrence estuary, one each from invasive (Atlantic clade, Fig. 1a) and noninvasive clades (North Atlantic clade, Fig. 1b). Survival was measured as (A) %hatching, (B) %survival from hatching to metamorphosis, and (C) %survival from metamorphosis to adult (also shown in Fig. 5). Treatments consisted of three food concentrations: (a) 700, (b) 2800, and (c) 14000 cells/mL in fresh water

Food Concentration (cells/mL)	(a)	(b)	(c)
	700	2800	14 000
**A. %Hatching**			
Montmagny (invasive Atlantic clade)	97.5 ± 2.5	95.0 ± 3.3	97.5 ± 2.5
St. Jean Port Joli (noninvasive N. Atlantic clade)	82.5 ± 8.8	92.5 ± 5.3	90.0 ± 5.3
Wilcoxon's *W* (*P*-value)	58.50 (0.20)	67.00 (1.00)	59.50 (0.45)
*t*-value from Ordinal Probit Model (*P*-value)	2.12 (**0.054)**	1.39 (0.19)	1.28 (0.22)
**B. %Survival from Hatching to Metamorphosis**			
Montmagny (invasive Atlantic clade)	65.0 ± 11.8	80.0 ± 6.5	77.5 ± 11.6
St. Jean Port Joli (noninvasive N. Atlantic clade)	30.0 ± 11.3	72.5 ± 8.4	57.5 ± 17.1
Wilcoxon's *W* (*P*-value)	50.00 (**0.046**)	64.00 (0.79)	63.00 (0.76)
*t*-value from Ordinal Probit Model (*P*-value)	2.97 (**0.011**)	2.56 (**0.024**)	2.38 (**0.033**)
**C. %Survival from Metamorphosis to Adult**			
Montmagny (invasive Atlantic clade)	32.5 ± 8.4	77.5 ± 5.9	75.0 ± 11.8
St. Jean Port Joli (noninvasive N. Atlantic clade)	0	32.5 ± 11.3	55.0 ± 16.4
Wilcoxon's *W* (*P*-value)	44.00 (**0.007**)	45.00 (**0.015**)	61.00 (0.49)
*t*-value from Ordinal Probit Model (*P*-value)	2.54 (**0.025**)	2.97 (**0.011**)	2.92 (**0.012**)

Values for survival are mean ± SE for *N* = 8 clutches. Statistical significance for difference in survival between the populations at each treatment was determined using the Wilcoxon rank sum test and a sequential ordinal probit model, which accounts for cumulative survival across life-history stages (*t*-values below, df = 13; see Materials and methods). Significant values (*P* < 0.05) are shown in bold. These comparisons were performed as *a priori* planned comparisons, and did not require multiple testing correction (Sokal and Rohlf 1995).

## Discussion

Saline to freshwater transitions have become increasingly common in recent years, with a large number of brackishwater species successfully invading freshwater lakes disproportionate to their rate of transport (Jażdżewski 1980; Lee and Bell 1999; Ricciardi and MacIsaac 2000; May et al. 2006; Ricciardi 2006; Lee and Gelembiuk 2008; Keller et al. 2011). The current practice of mid-ocean ballast exchange is likely to further bias freshwater invasions toward more brackishwater species, as they would be more likely to survive offshore ballast water exchanges (Ricciardi 2006; Ellis and MacIsaac 2009). The preponderance of freshwater invasions by brackish species is remarkable, given that brackishwater species tend to be physiologically maladapted freshwater conditions (Taylor 1985; Taylor and Harris 1986; Dietz et al. 1996; Lee and Petersen 2003; Lee et al. 2003), and have been found to undergo rapid evolution to tolerate freshwater environments (Lee et al. 2003, 2007, 2011, 2012). Salinity and food concentration tend to differ greatly between the native and invaded ranges of these invaders, with the native estuarine zone typically characterized by higher salinity and food concentrations (∼10 000 to >200 000 cells/mL) (Lovejoy et al. 1993; Vincent et al. 1994; Martineau et al. 2004) relative to the low salinity and lower food concentrations of many of the invaded lakes (Great Lakes: 250–1500 cells/mL) (Makarewicz 1993). Previous studies have established the importance of salinity in limiting invasions from saline into freshwater habitats (Taylor 1985; Taylor and Harris 1986; Dietz et al. 1996; Lee et al. 2003, 2007, 2011, 2012). However, this study is the first to examine the role of food concentration, as well as the interaction between food concentration and salinity tolerance, in facilitating saline to freshwater invasions.

### High food quantity facilitates freshwater invasions by saline populations

This study revealed the evolution of physiological response to food concentration and salinity following the invasion from saline to freshwater habitats. The freshwater invading population evolved the ability to tolerate low-food and low-salinity (freshwater) conditions to a significantly greater degree than its brackishwater ancestor (Figs 2A and 3; significant *Population* × *Salinity* and *Population* × *Food* in Tables 1 and 2A). This result was consistent with adaptation to freshwater environments, which are depauperate in ions and often food-poor relative to saline habitats. Lower reliance on high food by the freshwater population would likely have evolved due to the evolution of other compensating mechanisms to tolerate freshwater conditions, such as mechanisms to prevent ion loss (i.e., lower integument permeability). Our results also revealed that high food concentration enhanced survival at the lower salinity (freshwater treatment 75 μS/cm) more than at higher salinity (1 PSU) (significant *Food* × *Salinity*, Table 1; Figs 2A and 3). Specifically, at low salinity, genotypes in the saline populations that exhibited reaction norms with low survival under low food conditions experienced much higher survival under high food conditions (Fig. 3A). Greater benefits of higher food concentration at the lower salinity would be expected, given the high energetic costs of ionic regulation at lower salinities (see last section below).

Together, these results revealed that high food concentration had greater beneficial impacts on survival for the ancestral saline population, particularly at lower salinity. In addition, this beneficial effect declined in the freshwater population relative to its saline ancestor (Fig. 4, see Results). These results point to high food concentration as a critically important factor for the saline population to expand its range into freshwater habitats. In short, higher food concentration could extend the range limits of more saline species by increasing survival under low-salinity conditions, enabling the invasion of freshwater and allowing subsequent freshwater adaptation to take place.

Thus, high food concentration could serve as an equalizer, in allowing species of saline ancestry, such as zebra mussels, to colonize novel freshwater environments and compete with resident freshwater species. Although species from more saline habitats would tend to be competitively inferior under low-salinity conditions (<1 PSU), ample food might allow the more saline species to not only co-exist with freshwater species, but in some cases to outcompete them. With sufficient food, saline species tend to grow faster and have higher fecundity relative to comparable freshwater species (Anger 1995; Peterson 2001). This phenomenon might be viewed as a type of ‘condition-specific competition’ (Dunson and Travis 1991; Taniguchi and Nakano 2000), where gradients in food and salinity could allow brackish invaders and freshwater residents to co-exist. For example, the North American Great Lakes tend to be more eutrophic along the coastlines (Holland and Beeton 1972), where the originally brackish copepod *E. affinis* tends to persist (Patalas 1972; Roth and Stewart 1973; Robertson and Gannon 1981).

Such a mechanism might help explain the proliferation in recent years of invaders from brackish habitats into freshwater lakes and reservoirs (Jażdżewski 1980; Lee and Bell 1999; Ricciardi and MacIsaac 2000; Cristescu et al. 2003, 2004; May et al. 2006; Ricciardi 2006; Lee and Gelembiuk 2008; Keller et al. 2011). Anthropogentic nutrient inputs might be facilitating this phenomenon, including into the increasingly eutrophied North American Great Lakes (Beeton 1965; Patalas 1972).

Significant differences in salinity tolerance between ancestral saline and freshwater invading populations were consistent with findings of previous studies (Lee et al. 2003, 2007), even though the prior studies used a saline population from a more saline portion of the estuary (from Baie de L'Isle Verte, at 5-40 PSU). However, in contrast to previous results (Lee et al. 2003, 2007), evolutionary tradeoffs between low- and high-salinity tolerance were not evident in this study. In the prior studies, an increase in freshwater tolerance during freshwater invasions was accompanied by a loss of high-salinity tolerance, consistent with tradeoffs (Lee et al. 2003, 2007). Tradeoffs might have not been manifested in this study because of the narrow range of salinities used (0 vs 1 PSU) relative to the much broader range of salinities used in previous studies (0 vs 25 PSU). In addition, unlike previous studies, this study found no significant differences in development time between the brackish and freshwater populations (Table 2B) (Lee et al. 2003, 2007). The prior studies speculated that tradeoffs might exist between low-salinity tolerance and development time. Again, the salinity contrast between the populations used in this study might have been too small for this evolutionary tradeoff to manifest.

The clear evolutionary shifts found for survival (Figs 2A and 3) were inconsistent with the lack of significant effects of *Clutch* (proxy for genotype) on survival, using the Lake Michigan and St. Lawrence (at Montmagny) populations (Table 1). Given the evolutionary shifts evident in this study, genetic variation would have to exist in the ancestral saline population (St. Lawrence at Montmagny) for physiological response to evolve. The lack of significance of *Clutch* deviated from prior studies (Lee et al. 2003, 2007) and even with the second experiment performed in this study (Table 3). It is likely that the lack of significance of clutch effects found here (Table 1) was due to lack of statistical power, especially given that significance was found for the same saline population (St. Lawrence at Montmagny) in the second experiment of this study (Table 3). Significant effects of *Clutch* (*P* < 0.0001) and *Clutch* × *Food* (*P* < 0.0001) on development time (Table 1B) indicated that development time and its plastic response to food concentration are heritable traits that could evolve, as found in previous studies (Lee et al. 2003, 2007).

### Differential impacts of food concentration on saline populations from the native range

This study revealed differences in the effect of food concentration on freshwater survival between saline populations from the native range, potentially revealing functional contrasts that might account for differences in invasive success among native range populations. The two saline populations, occurring in close proximity in the estuarine transition zone (Fig. 1a,b), exhibited striking contrasts in freshwater survival at low food concentration, with significantly greater survival in the invasive clade population (Fig. 1a, Montmagny) than in the noninvasive clade population (Fig. 1b, St. Jean Port Joli) (Fig. 5, Table 4). Most notable was the complete lack of survival to adulthood under low food conditions in the noninvasive clade population (Fig. 5C, Table 4C), suggesting that insufficient food concentration could pose a key barrier to range expansions into freshwater habitats for certain populations more than others. Thus, while the ancestral saline population (St. Lawrence at Montmagny) required significantly higher food concentration to tolerate low salinities than the freshwater Lake Michigan population (Fig. 2A) (previous section), the saline population from the noninvasive clade (St. Lawrence at St. Jean Port Joli) required even more (Fig. 5). The significant effects of *Clutch* (*P* = 0.005) and *Clutch* × *Food* (*P* = 0.0003) on survival (Table 3) revealed the presence of genetic variation in survival and the potential for response to food concentration to evolve.

When cumulative survival across all life-history stages was taken into account (using an ordinal probit model, see Materials and methods), the invasive clade population showed significantly greater survival to juvenile (Table 4B) and adult stages (Table 4C) across all food concentrations. This result does indicate that the invasive clade population would outcompete the noninvasive clade population under freshwater conditions at all food concentrations tested. However, the total lack of survival at the lowest food concentration (700 cells/mL) in the noninvasive clade population indicates that food concentration is clearly a limiting factor for this population (Fig. 5C). In addition, the trend toward greater survival to adulthood at higher food concentrations for the noninvasive clade population (Fig. 5C, blue line) suggests that even higher food concentrations might provide a greater ‘rescue effect’ in enhancing freshwater survival for this population.

Within the past ∼60 years, the Atlantic clade has given rise to freshwater invading populations multiple times independently along the Atlantic coast, whereas the North Atlantic clade conspicuously has not (Lee 1999; Winkler et al. 2008). While the Atlantic clade has colonized completely fresh water (0 PSU salinity), the lower limit for salinity distribution of the North Atlantic clade is 0.1–0.3 PSU (G. Winkler, unpublished data). Differences in osmoregulatory capacity and energetic requirements might cause fresh water to pose a more acute barrier for some populations and clades relative to others. For example, cost of growth remains relatively constant across a wide salinity range for the copepod *Acartia tonsa*, but increases dramatically for its congener *A. clausi* at lower salinities (Calliari et al. 2006).

Although our results are insufficient to draw general conclusions regarding evolutionary differences between the invasive and noninvasive clades, distribution patterns of the two clades suggest that selection regime in the native range might be a factor (Lee and Gelembiuk 2008; Winkler et al. 2008; Hufbauer et al. 2012). Recent theoretical and empirical studies suggest that selection regimes within the native ranges of invasive populations might be important for creating conditions under which invasive potential could evolve (reviewed in Lee and Gelembiuk 2008). For instance, fluctuating environments might give rise to populations with enhanced ability to invade by selecting for generalist strategies or greater evolutionary potential (Lee and Gelembiuk 2008).

Heterogeneity of microhabitats within the native range might lead to differences in physiology, as well as evolutionary and invasive potential. While the two clades overlap in distribution in their native range, fine-scale differences exist between the habitats where each clade predominates. Populations of the noninvasive North Atlantic clade tend to predominate in the central region of the estuarine transition zone (Winkler et al. 2008), characterized by consistently high phototrophic productivity supporting high standing stocks of zooplankton (Vincent et al. 1996). In contrast, invasive (Atlantic) clade populations tend to be more prevalent along the margins of the estuarine transition zone, predominating at the head of the estuarine transition zone and also in the more downstream salt marshes, where they cannot escape large fluctuations in salinity (Winkler et al. 2008). An evolutionary history in these marginal habitats, removed from the food-rich center of the estuarine transition zone and exposed to fluctuating salinity, might have selected for the physiological capacity to colonize and survive under low-food and low-salinity conditions.

In addition, the noninvasive clade population (Fig. 1b) was collected 41 km downstream from the invasive clade population (Fig. 1a), such that that prior adaptation to higher salinity might have contributed to the greater need of high food for freshwater survival. Prior adaptation to higher salinity might have resulted in higher integument permeability and greater ion efflux, resulting in a greater need for ion uptake and increased energetic costs (and greater need for food) under freshwater conditions.

Systematic comparisons of multiple populations from both the invasive and noninvasive clades from their overlapping range in the estuary would allow us to determine much more conclusively whether functional differences exist between the clades, and which factors might have contributed to those differences. Such a comparison would be required to determine whether evolutionary history might have led to adaptive differences between the clades that affect their ability to colonize novel environments.

### Food concentration as a key factor enabling freshwater invasions

So then, why would higher food concentration enhance the ability of the saline populations to tolerate freshwater conditions? Food quantity (and quality) might be critically important for extending the physiological limits of brackish water species by satisfying the increased energetic demands of ionic regulation under freshwater conditions. For both fresh and saline populations of *E. affinis,* demands for ionic regulation increase under freshwater conditions, where ion uptake must increase and hemolymph concentrations must be maintained at elevated levels relative to the environment (Lee et al. 2011, 2012). These functions are energetically costly, as they entail ion transport and maintenance of ionic concentrations against very steep concentration gradients (Willmer et al. 2004).

Relative to the saline populations, the freshwater populations exhibited evolutionary shifts toward higher levels of ion uptake and higher hemolymph concentrations (osmolalities) in freshwater environments (Lee et al. 2011, 2012). However, freshwater species and populations are likely to have acquired adaptations that would make them energetically more efficient in fresh water (e.g., reduced ion efflux, more efficient metabolism), relative to saline populations naive to freshwater conditions. Lack of such freshwater adaptations would make the energetic demands on saline species exceedingly high, requiring very high food intake to initially colonize freshwater habitats and survive prior to evolutionary adaptation.

Yet, despite evidence that the freshwater populations have evolved greater freshwater tolerance relative to their saline ancestors (Lee et al. 2003, 2007, 2011, 2012), many of these recent immigrants appear to still require high levels of food relative to more ancient freshwater species. This need for more food might reflect the fact that the freshwater invaders are still undergoing the process of freshwater adaptation and might require excess food for ionic regulation in fresh water. For instance, in the Great Lakes of North America, *E. affinis* tends to inhabit eutrophic nearshore environments within the lakes, such as the highly eutrophic Racine Harbor and Green Bay in Lake Michigan (CE Lee, pers. obs.) (Patalas 1972; Roth and Stewart 1973; Gannon 1974). The zebra mussel *Dreissena polymorpha*, which originated from the brackish waters of the Ponto-Caspian basin, is notorious as a voracious consumer of algae with exceedingly high filtration rates in freshwater habitats (Fanslow et al. 1995; Parker et al. 1998) and is estimated to have higher growth rates in more eutrophic environments (Schneider 1992). The zebra mussel displays an unusually leaky epithelium and suffers high ionic losses relative to native freshwater bivalves (Dietz et al. 1996). Such ionic losses are likely the reason that zebra mussels exhibit higher ion transport rates than native freshwater bivalves (Dietz et al. 1996). Similarly, the amphipod *Corophium curvispinum*, also from the brackish Ponto-Caspian region (Jażdżewski 1980), has been speculated to have successfully invaded the Lower Rhine River as a result of eutrophication and industrial discharges (van den Brink et al. 1993). This amphipod is also an inefficient osmoregulator in fresh water and requires Na^+^ levels above 0.5 mM for osmoregulation (Taylor and Harris 1986). Thus, in addition to facilitating initial stages of invasions from saline to freshwater habitats, high food concentrations might also be crucial for maintaining the new immigrants as longer term residents in freshwater habitats, during which the processes of freshwater adaptation could proceed further.

In this study, we tested the hypothesis that food amount is important for freshwater survival by species of brackish origin, as previous studies had already established that food type is critical (Vanderploeg et al. 1996; Lee et al. 2003; Naddafi et al. 2007). For freshwater survival, the copepod *E. affinis* and zebra mussel larvae require high concentrations of algae rich in particular long-chain polyunsaturated fatty acids (such as the cryptophyte *Rhodomonas minuta*) (Ahlgren et al. 1992; Vanderploeg et al. 1996; Lee et al. 2003). In addition, freshwater populations of *E. affinis* and zebra mussels are typically found in the presence of cryptophytes in nature (Munawar and Munawar 1976; Wehr and Thorp 1997; Lee 1999; Naddafi et al. 2007). Cryptophytes comprise 6-24% of the phytoplankton mass in Lake Erie (total biomass is 1.5–7 g/m^3^) (Munawar and Munawar 1976), where many brackishwater species have invaded successfully (Lee 1999; Lee and Bell 1999; Ricciardi and MacIsaac 2000). In contrast to freshwater populations, populations of *E. affinis* residing in saline water could survive and reproduce on a much broader diet (Heinle and Flemer 1975; Simenstad et al. 1990; Baross et al. 1994). In future studies, it would be worth exploring which particular dietary nutrients provided by cryptophytes are essential for freshwater survival by brackish invaders, as it would help identify the specific physiological mechanisms underlying constraints on freshwater invasions. Moreover, it would be worth investigating the types of anthropogenic nutrient inputs that favor particular species of algae, with potential consequences for favoring brackish invaders relative to others.

In conclusion, our results suggest that high concentrations of food (of particular types) could enable more saline invaders to overcome a formidable biogeographic barrier and greatly extend their range limits into freshwater habitats. Increases in anthropogenic nutrient inputs in recent years might be extending physiological limits of brackishwater species, in allowing them to invade and subsequently evolve in otherwise uninhabitable freshwater habitats. This study highlights the fact that insights into physiological limits of invasive populations, and how such limits could evolve, could help us predict the potential of these populations to extend their ranges into novel habitats.

## Data archiving statement

Data for this article are available at: Dryad: doi:10.5061/dryad.gd0hv.
